# A serological survey of wild boar in Serbia for *Mycoplasma hyopneumoniae* and *Actinobacillus pleuropneumoniae*

**DOI:** 10.17221/64/2024-VETMED

**Published:** 2024-12-27

**Authors:** Milan Ninkovic, Jelena Maksimovic Zoric, Dragica Vojinovic, Ljubisa Veljovic, Nemanja Jezdimirovic, Jasna Kureljusic, Jadranka Zutic

**Affiliations:** Scientific Institute of Veterinary Medicine of Serbia, Belgrade, Serbia

**Keywords:** antibodies, ELISA, pneumonia, Serbia, wild swine

## Abstract

*Mycoplasma hyopneumoniae* and *Actinobacillus pleuropneumoniae* are causative agents of the porcine respiratory disease complex. However, information on the prevalence of these pathogens in wild boars is scarce. This study aimed to investigate the presence of antibodies to *Mycoplasma hyopneumoniae* and *Actinobacillus pleuropneumoniae* in wild boars in Serbia. In this study 253 serum samples from wild boars were tested for antibodies to *Mycoplasma hyopneumoniae* and *Actinobacillus pleuropneumonia* using the ELISA assay. The overall seroprevalence rates of *Mycoplasma hyopneumoniae* and *Actinobacillus pleuropneumoniae* were 4.2% and 56.9%, respectively. Antibodies to both pathogens were detected in 20 sera samples (7.9%). The prevalence of wild boars that were seropositive for *Mycoplasma hyopneumoniae* differed with age and ranged from 10.7% to 33.3%, and for *Actinobacillus pleuropneumoniae,* it ranged from 51.8% to 83.3%. Wild boars are hard to control and are considered a high-risk infection source for outdoor and backyard pigs and eventually for commercial indoor farms as well. Thus, the result of this first serosurvey in Serbia should raise awareness of the importance of wild boars as potential reservoirs of bacterial pathogens such as *Mycoplasma hyopneumoniae* and *Actinobacillus pleuropneumoniae*. Our data revealed the circulation of both pathogens in wild boars in Serbia, drawing attention to the potential health risk they present for domestic swine health.

There are numerous reports of an association between wild boars and domestic swine in the maintenance and transmission of diseases (Elbers et al. 2000; Marinou et al. 2015). Wild boars are important reservoirs for various pathogens (Vengust et al. 2006). Previous studies have shown that wild boar can carry pathogens such as African swine fever, *Actinobacillus pleuropneumoniae* (APP), and *Mycoplasma hyopneumoniae* (MHYO), causative agents of some of the most important health problems within the domestic swine farming sector across Europe (Vengust et al. 2006; Marinou et al. 2015; Prodanov-Radulovic et al. 2023). MHYO and APP are described as the primary causes of pneumonia and pleuropneumonia, one of the major health issues in swine production worldwide. Currently, 19 different serotypes of APP are described in domestic swine (Stringer et al. 2021). The main route of their transmission is direct contact or aerosol (Torremorell et al. 1997). The clinical manifestation of the disease in domestic swine depends on several factors, such as the pathogenicity of the APP strain, the presence of co-infections, the immune status of the herd, and the individual immune response (Rycroft and Garsiede 2000). APP may cause acute disease with high mortality; however, generally, the disease has a chronic onset with few clinical signs and a low mortality. Even so, the presence of this pathogen in a swine herd is followed by high economic losses related to lowered production and increased medical costs (Sassu et al. 2017). However, in wild boars, the clinical manifestations and pathological changes caused by APP are rarely present (Reiner et al. 2010).

*Mycoplasma hyopneumoniae* is considered a primary agent that causes enzootic pneumonia and leads to the development of chronic respiratory disease (Maes et al. 2008). In swine, MHYO induces a continuous inflammatory reaction and immunosuppression, facilitating infection with other pathogens involved in the porcine respiratory disease complex (PRDC) (Leal Zimmer et al. 2020). Chronically infected pigs are the source of infection for other susceptible animals, and transmission occurs horizontally and vertically. The control of MHYO infection in industry production is complex due to MHYO’s infection dynamics (intracellular opportunistic microorganisms), antimicrobial resistance, and strain diversity (Maes et al. 2008).

MHYO infections in wild boars have been reported in European countries, such as Spain and Slovenia (Sibila et al. 2010; Stukelj et al. 2014), while APP has been confirmed in Germany, Finland, and Slovenia (Reiner et al. 2010; Halli et al. 2014; Stukelj et al. 2014). Investigations into these two pathogens in Serbia have been mostly related to pig farms (Zutic et al. 2014), where they present some of the most significant infectious agents involved in PRDC aetiology (Savic et al. 2015). However, the information about the circulation of these pathogens among wild boars is scarce (Prodanov-Radulovic et al. 2015).

Wild boars are territorial animals when they have enough food; however, they can cross long distances when food is lacking (Tarvydas and Belova 2022). Due to these movements, there is a risk of spreading pathogens to new territories and domestic swine populations. Swine production systems characterised by inadequate biosecurity measures, poor husbandry practices, and the raising of free-roaming domestic pigs located near high-density wild boar areas increase the probability of the spillover of different pathogens (Prodanov-Radulovic et al. 2015; Makovska et al. 2023), and these have been widely distributed across Serbian territory (Prodanov-Radulovic et al. 2015; Neskovic et al. 2021). Therefore, this study aimed to investigate the circulation of these two pathogens in the wild boar population and determine the seroprevalence of APP and MHYO in wild boars in Serbia.

## MATERIAL AND METHODS

Ethical review and approval were waived for this study since the animals were hunted and sampled during the hunting season regulated in the Law on Game and Hunting, Official Gazette of the Republic of Serbia No. 18/10 (Ministry of Agriculture, Forestry and Water Management 2010), and no animals were killed for research purposes.

### Sample collection and data collection

**Figure 1:**
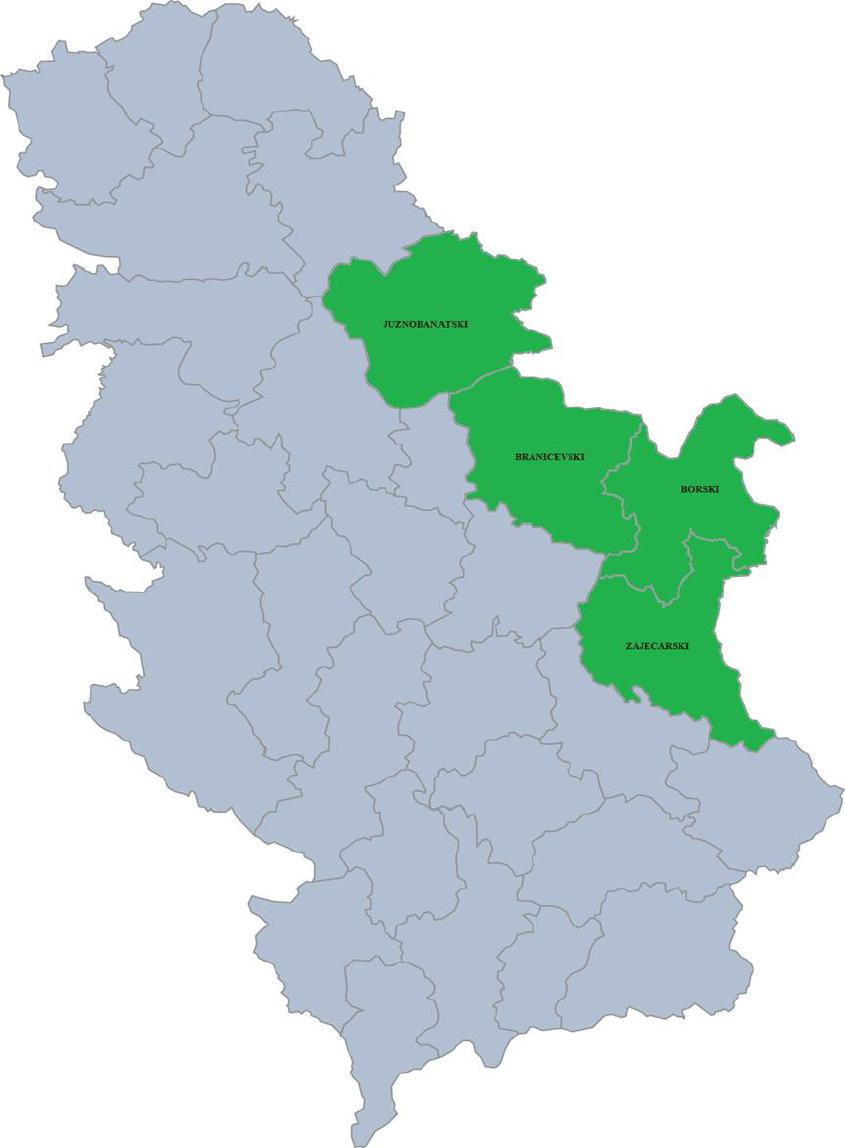
Administrative districts in Serbia where the tested samples were collected

The samples originated from wild boars hunted during the 2022 hunting season in the territory of four administrative districts: Juznobanatski, Branicevski, Borski, and Zajecarski districts (Figure 1).

Officially designated veterinarians collected the sera as a part of the surveillance program for classical and African swine fever in wild boars in Serbia [Plan and method of monitoring of classical swine fever and African swine fever in wild boars in 2022, No. 323-02-11961/2022-05 (Ministry of Agriculture, Forestry and Water Economy 2023)]. Blood from the shot animals was collected into sterile tubes and sent to the laboratory. Serum was obtained by centrifugation at 400 × *g* for 10 min and then frozen at –20 °C until examination. Data on the age category, sex, and location of the hunted animal accompanied each sample. The age category was determined according to the number of permanent molars (SCHEDA Ecological Associates, Inc), and all animals were classified into one of four age groups: without molars, 0–6 months; one molar, 7–18 months; two molars, 19–30 months; three molars, 31+ months. According to the Statistical Office of the Republic of Serbia (2021), the number of wild boars in Serbia in 2021 was 23 856. In this study, a total of 253 wild boar sera samples were tested for the presence of antibodies (Abs) to APP and MHYO. Of these, 141 (55.7%) originated from males and 112 (42.3%) from females.

### Detection of Abs to *Actinobacillus pleuropneumoniae*

A commercial ELISA ID Screen^®^ APP Screening Indirect kit (IDVET, Montpellier, France) was used to detect Abs. According to the manufacturer’s internal validation report, this test is characterised by good analytical specificity (ASp) and excellent diagnostic specificity (DSp) (DSp = 95%, CI = 97.7–100%). Based on the validation report, the analytical sensitivity (ASe) for serotypes 1 to 12 was 100%, and the diagnostic sensitivity (DSe) was estimated by examining the sera from sick animals and confirmed by testing the same samples with serotyping kits. The samples and controls were tested at a final dilution of 1 : 200 according to instructions in the user’s manual. The optical density (OD) of the tested samples was read at 450 nm using a Tecan sunrise reader (Tecan Group LTD, Männedorf, Switzerland). The sample-to-positive ratio (S/P) was calculated according to the formula prescribed in the user’s manual, and samples with an S/P of ≤ 25%, between 25% and 30%, and ≥ 30% were categorised as negative, doubtful, and positive, respectively.

### Detection of Abs to *Mycoplasma hyopneumoniae*

For the detection of Abs to MHYO, a commercial ELISA ID Screen^®^ *Mycoplasma hyopneumoniae* Competition kit (IDVET, Montpellier, France) was used. According to the manufacturer’s validation report, this test is characterised by good ASp and excellent DSp (DSp = 95%, CI = 99.7–100%). The ASe and DSe are concordant with the values of other widely used ELISA kits. The samples and controls were tested at a dilution of 1 : 2, according to the instructions in the user’s manual. The optical density of each tested sample was read using a Tecan sunrise reader (Tecan Group LTD, Männedorf, Switzerland) at 450 nm. The obtained ODs were used to calculate the sample-to-negative ratio (S/N) following the formula in the user’s manual. Samples with S/N > 50% were regarded as negative, and the ones with S/N ≤ 50% were considered positive for MHYO Abs.

### Statistical analysis

The data were analysed using descriptive statistics regarding the territorial distribution, age, and sex of the examined individuals. The confidence intervals were calculated using the Wilson score method. For better understanding, multinomial logistic analysis was conducted by taking the disease as the outcome (separately for APP, MHYO, or both) and the healthy group as the reference. The factors for the disease outcome were the age and district with a sex covariate. The reference point was set in the 7–18 months old group. The sex reference point was set to males, and the district reference category was randomly set to the Borski district using IBM SPSS v21 (Armonk, USA).

## RESULTS

Out of a total of 253 sera, Abs were detected to APP in 144 animals (56.9%; 95% CI: 50.7% to 62.8%) and to MHYO in 36 animals (14.2%; 95% CI: 10.4% to 19.1%), while 20 animals possessed Abs to both pathogens (7.9%; 95% CI: 4.8% to 12.2%).

Regarding the territorial distribution, the APP and MHYO seroprevalences are shown in Table 1. Based on the positive-to-tested per district, the seroprevalence for APP ranged from 50.5% in the Branicevski to 65.5% in the Juznobanatski district. The highest seroprevalence for MHYO was 22.4%, in the Borski district (Table 1). Out of 36 MHYO-seropositive wild boars, 20 (55.6%) had Abs to APP as well.

**Table 1:** The APP and MHYO seroprevalence according to districts

Distribution of positive sample	Total	APP	MHYO	APP + MHYO
By district (*n*)		positive	positive	positive
Juznobanatski	*n* = 84	55 (65.5%)	11 (13.1%)	7 (8.3%)
Branicevski	*n* = 46	23 (50.5%)	3 (6.5%)	3 (6.5%)
Borski	*n* = 58	32 (55.2%)	13 (22.4%)	6 (10.3%)
Zajecarski	*n* = 65	34 (52.3%)	9 (13.8%)	4 (6.2%)

APP = *Actinobacillus pleuropneumoniae*; APP + MHYO = *Actinobacillus pleuropneumoniae* + *Mycoplasma hyopneumoniae*; MHYO = *Mycoplasma hyopneumoniae*

The MHYO seroprevalence in the different age groups ranged from 10.7% to 33.3% and the APP from 51.8% to 83.3% (Table 2). The highest MHYO and APP seroprevalences (33.3% and 83.3%, respectively) were noted in the 0–6-month-old wild boars, as well as MHYO and APP co-infections (Table 2).

**Table 2:** The APP and MHYO seroprevalence according to age categories

Age of months	Total	APP *n* (%)	MHYO *n* (%)	APP + MHYO *n* (%)	Negative *n* (%)
0–6	*n* = 6	5 (83.3%)	2 (33.3%)	1 (16.6%)	0 (0%)
7–18	*n* = 112	58 (51.8%)	12 (10.7%)	5 (4.5%)	47(42.0%)
19–30	*n* = 78	46 (59.0%	12 (15.4%)	9 (11.5%)	29 (38.1%)
31+	*n* = 57	35 (61.4%)	10 (17.5%)	5 (8.8%)	17 (29.8%)

APP = *Actinobacillus pleuropneumoniae*; APP + MHYO = *Actinobacillus pleuropneumoniae* + *Mycoplasma hyopneumoniae*; MHYO = *Mycoplasma hyopneumoniae*

When the obtained results were assorted by sex, the seropositivity for MHYO was more common in females than in males (17.9% vs 11.3%), while the seropositivity for APP was higher in male individuals (62.4%). Both MHYO and APP Abs were detected at a slightly higher rate in female animals (8.0%) (Table 3).

**Table 3:** The APP and MHYO seroprevalence according to sex

Sex	Total	APP *n* (%)	MHYO *n* (%)	APP + MHYO *n* (%)	Negative *n* (%)
Male	*n* = 141	88 (62.4%)	16 (11.3%)	11 (7.8%)	48 (34.0%)
Female	*n* = 112	56 (50.0%)	20 (17.9%)	9 (8.0%)	45 (40.2%)

APP = *Actinobacillus pleuropneumoniae*; APP + MHYO = *Actinobacillus pleuropneumoniae* + *Mycoplasma hyopneumoniae*; MHYO = *Mycoplasma hyopneumoniae*

The multinomial logistic analysis final model performed well with *P* = 0.016 (Chi = 37.148, DF 21). The performance of the analysis is provided in the Electronic Supplementary Material (ESM) Tables S1, S2, and S3. The age groups showed a declining risk factor of protracting seropositivity with all age groups above 6 months, but the beta coefficients in age groups older than 6 months were similar. The analysis according to the geographic distribution showed different serology outcomes in different districts. In the Borski district, animals with Abs to MHYO or both diseases were dominant, while in the Juznobanatski, the majority of the animals tested possessed Abs to APP. The difference according to the geographic distribution was numerical and statistically insignificant (*P* > 0.05).

## DISCUSSION

The high incidence of wild boars with Abs to APP and MHY, two microbes commonly involved in the PRDC, raises concern since wild boars have been suggested as a potential reservoir of these bacterial pathogens and a possible source of infection for domestic swine (Vengust et al. 2006; Touloudi et al. 2015). In Serbia, Zutic et al. (2014) reported relatively low APP seroprevalence in the categories of sows and gilts (12.6%), which could be related to the implementation of biosecurity protocols aimed at the suppression of the spread of pathogens such as APP and MHYO (Bojkovski et al. 2018). Contrary to this, Pepovich et al. (2022) and Angjelovski et al. (2023) reported higher overall APP seroprevalence (above 50%) in commercial pig farms in Bulgaria and North Macedonia. Despite the importance of these two pathogens in commercial pig farms, few works have been undertaken on their status and distribution in wild boar populations. In Europe, Slovenia reported a seroprevalence of 52% amongst hunted wild boars, while Finland and Greece reported a seroprevalence of 12.6% and 90.5%, respectively, in farmed wild boars (Vengust et al. 2006; Halli et al. 2014; Marinou et al. 2015). Our results are similar to those of Vengust et al. (2006) and reveal the circulation of these pathogens in the wild boar population. Two possible paths of APP transmission between wild boar populations exist; close contact with infected domestic swine or infected wild boars from other territories (Sassu et al. 2017). Based on our results and the pathogen characteristics, we assume that, in Serbia, APP transmission occurs and is maintained within wild boar populations by direct contact or aerosol transmission and suggest that the wild boars present a reservoir of infection for free-ranging backyard pigs, usually reared in Eastern Serbia and the Vojvodina province (Prodanov-Radulovic et al. 2015; Neskovic et al. 2021).

Analysing sex as a risk factor showed that males are more prone to being diagnosed with APP than females. Contrarily, MHYO or both diseases combined are more prone to being diagnosed in females. Regarding sex, the *P*-value was less than 0.05 only concerning APP.

Regarding MHYO infection in domestic swine in Serbia, a high seroprevalence (88.0%) has been reported in pigs from farrow-to-finish commercial farms (Prodanov-Radulovic et al. 2020). In Germany, which reported similar results, MHYO was present in 87.5% of the examined farms, with seroprevalence varying between 0% and 100% (Deffner et al. 2022). Unlike in domestic pigs, lesions in the lungs of wild boars related to the pathogenic activity of MHYO were rarely evident (Reiner et al. 2010; Stukelj et al. 2014; de Souza et al. 2021). They develop an inapparent infection without the clinical signs and pathomorphological changes, these facts indicate a minor impact of MHYO in wild boars than in conventionally farmed domestic pigs (Batista Linhares et al. 2015). That is why serological diagnostics is usually used when studying the MHYO infection in wild boars. In our study, Abs to MHYO were detected in 36 wild boar sera (14.2%). A similar study was also performed in Slovenia, which resulted in 15.8% seropositivity in wild boars (Stukelj et al. 2014). In Spain, analogous results were obtained with the MHYO seroprevalence at 21.0% and 13.9%, respectively (Sibila et al. 2010; Risco et al. 2014). However, several countries reported a significantly higher seroprevalence for MHYO in wild boar populations in contrast to our results. In Brazil, de Souza et al. (2021) discovered a seroprevalence of 65.9%, while Marinou et al. (2015) reported Abs to MHYO in 72.5% of the tested wild boars in Greece. These results are related to cross-breeding between wild boars and free-ranging pigs or local domestic breeds (Marinou et al. 2015).

Our results prove the circulation of MHYO in wild boars is characterised by a lower incidence of seropositivity than in the population of domestic pigs reared in commercial farms (Prodanov-Radulovic et al. 2020). The large difference in seroprevalence between domestic and wild pigs is likely due to different breed-related susceptibilities to MHYO (Liang et al. 2018). Based on the low seroprevalence, we suggest that wild boars are not reservoirs of this pathogen for the domestic swine population. The presence of MHYO in the swine body and its immuno-suppressive influence present predisposing factors for infections with other pathogens such as APP and *Pasteurella multocida*, and the occurrence of the PRDC (Zdravkovic et al. 2023). Our results support this, where 55.6% of MHYO-seropositive wild boars had Abs to APP. There are scarce literature data regarding the serological investigation of both; however, Marinou et al. (2015) reported a significantly higher seroprevalence in wild boars in Greece (44.6%).

Regarding the age category, our results suggest that the highest seroprevalence of MHYO and APP boars was noted in the 0–6 months age category. However, these results must be considered with caution, bearing in mind that our study included about 1% of the population of wild boars in Serbia and that the number of 0–6-months-old tested pigs was very low (only six animals). Concerning sex, our findings indicate the presence of a higher seroprevalence of MHYO in females than in males, which is consistent with the results of de Souza et al. (2021). Opposite to this, APP seroprevalence was higher in male wild boars. This could be related to the movements over long distances, more frequent close contact between and within groups, and easier airborne transmission.

In conclusion, this is the first report on MHYO and APP seroprevalence in wild boars in Serbia. The results revealed a higher overall seroprevalence of APP than MHYO. Our results showed that wild boars in Serbia are exposed to APP and MHYO; however, the seroprevalence fluctuates across districts. The results of this study can serve as a basis for future research on seroprevalence in outdoor and backyard pigs where biosecurity measures are scarce and there is a risk of contact with wild boars.

## Supplementary Files

Electronic Supplementary Material (ESM) Tables
