# Supplementary material for: A serological survey of wild boar in Serbia for Mycoplasma hyopneumoniae and Actinobacillus pleuropneumoniae
Source: Vet Med (Praha). 2024 Dec 27;69(12):413–9. doi: 10.17221/64/2024-VETMED (PMC11742286; doi:10.17221/64/2024-VETMED)
Supplement: Electronic Supplementary Material (ESM) Tables [file VETMED-69-12-124064-s001.pdf]

https://doi.org/10.17221/64/2024-VETMED

A serological survey of wild boar in Serbia for *Mycoplasma hyopneumoniae* and *Actinobacillus pleuropneumoniae*

Milan Ninkovic, Jelena Maksimovic Zoric, Dragica Vojinovic, Ljubisa Veljovic, Nemanja Jezdimirovic, Jasna Kureljusic, Jadranka Zutic*

Scientific Institute of Veterinary Medicine of Serbia, Belgrade, Serbia

**Corresponding author: milan.ninkovic1992@gmail.com*

The authors are fully responsible for both the content and the formal aspects of the electronic supplementary material. No editorial adjustments were made.

Electronic Supplementary Material (ESM)

Table S1. Model fitting information

Table S2. Likelihood ration test

Table S3. Parameter estimates in the multinomal logistic analysis

https://doi.org/10.17221/64/2024-VETMED

Table S1. Model fitting information

Model	Model fitting criteria			Likelihood ratio tests		
	AIC	BIC	–2 Log Likelihood	Chi-square	*df*	Sig.
Final	200.902	285.703	152.902	37.148	21	0.016

Table S2. Likelihood ration test

Effect	Model fitting criteria			Likelihood ratio tests		
	AIC of reduced model	BIC of reduced model	–2 Log Likelihood of reduced model	Chi-square	*df*	Sig.
Intercept	200.902	285.703	152.902	0.000	0	–
Sex	206.688	280.890	164.688	11.787	3	0.008
Age	194.162	247.163	164.162	11.261	9	0.258
District	200.499	253.499	170.499	17.597	9	0.040

The chi-square statistic is the difference in –2 log-likelihoods between the final model and a reduced model. The reduced model is formed by omitting an effect from the final model. The null hypothesis is that all parameters of that effect are 0

https://doi.org/10.17221/64/2024-VETMED

Table S3. Parameter estimates in the multinomal logistic analysis

Disease[a]		B	Std. error	Wald	*df*	Sig.	Exp(B)	95% Confidence interval for Exp(B)	
								lower bound	upper bound
APP	Intercept	18.035	1.409	163.803	1	0.000	–	–	–
	Sex	0.660	0.315	4.407	1	0.036	1.936	1.045	3.586
	[Age 6–18 months]	–18.747	1.297	208.938	1	0.000	7.218E-9	5.682E-10	9.170E-8
	[Age 1.5–2.5 years]	–18.780	1.295	210.406	1	0.000	6.979E-9	5.517E-10	8.827E-8
	[Age > 2.5 years]	–18.980	1.259	227.242	1	0.000	5.718E-9	4.848E-10	6.744E-8
	Age 0–6 months	0	–	–	0	–	–	–	–
	[District = Zajecarski]	–0.264	0.423	0.391	1	0.532	0.768	0.335	1.759
	[District = Branicevski]	–0.252	0.443	0.322	1	0.570	0.778	0.326	1.854
	[District =Junibanatski]	0.516	0.408	1.605	1	0.205	1.676	0.754	3.725
	[District = Borski]	0	–	–	0	–	–	–	–
MHYO	Intercept	20.321	1.913	112.856	1	0.000	–	–	–
	Sex	–1.109	0.614	3.255	1	0.071	0.330	0.099	1.101
	[Age 6–18 months]	–19.012	1.678	128.421	1	0.000	5.537E-9	2.066E-10	1.483E-7
	[Age 1.5–2.5 years]	–20.248	1.759	132.446	1	0.000	1.608E-9	5.113E-11	5.057E-8
	[Age > 2.5 years]	–19.559	1.622	145.475	1	0.000	3.203E-9	1.334E-10	7.689E-8
	Age 0–6 months	0	–	–	0	–	–	–	–
	[District = Zajecarski]	–1.011	0.731	1.916	1	0.166	0.364	0.087	1.523
	[District = Branicevski]	–19.180	4 522.173	0.000	1	0.997	4.680E-9	0.000	–
	[District =Junibanatski]	–1.081	0.730	2.194	1	0.139	0.339	0.081	1.418
	[District = Borski]	0	–	–	0	–	–	–	–
APP + MHYO	Intercept	18.452	1.147	258.957	1	0.000	–	–	–
	Sex	–0.133	0.561	0.056	1	0.813	0.875	0.291	2.630
	[Age 6–18 months]	–19.166	0.702	745.137	1	0.000	4.747E-9	1.199E-9	1.880E-8
	[Age 1.5–2.5 years]	–19.145	0.650	867.125	1	0.000	4.847E-9	1.356E-9	1.733E-8
	[Age > 2.5 years]	–20.065	0.000	–	1	0.000	1.932E-9	1.932E-9	1.932E-9
	Age 0–6 months	0	–	–	0	–	–	–	–
	[District = Zajecarski]	–0.660	0.774	0.726	1	0.394	0.517	0.113	2.358
	[District = Branicevski]	–0.904	0.794	1.297	1	0.255	0.405	0.085	1.919
	[District =Junibanatski]	–0.074	0.663	0.013	1	0.911	0.928	0.253	3.402
	[District = Borski]	0	–	–	0	–	–	–	–

[a]The reference category is: Healthy

APP = *Actinobacillus pleuropneumoniae*; APP + MHYO = *Actinobacillus pleuropneumoniae* + *Mycoplasma hyopneumoniae*; MHYO = *Mycoplasma hyopneumoniae*
